# Omega-3 Fatty Acids as Potential Predictors of Sudden Cardiac Death and Cardiovascular Mortality: A Systematic Review and Meta-Analysis

**DOI:** 10.3390/jcm14010026

**Published:** 2024-12-25

**Authors:** Ji Young Kim, So Yeon Joyce Kong, Eujene Jung, Yong Soo Cho

**Affiliations:** 1 Department of Medicine, Seoul National University, Seoul 08826, Republic of Korea; pedkjy@gmail.com; 2 Strategic Research, Laerdal Medical, 4002 Stavanger, Norway; soyeon.kong@gmail.com; 3 Department of Emergency Medicine, Chonnam National University Hospital, Gwangju 61469, Republic of Korea; 4 Department of Emergency Medicine, Chonnam National University Medical School, Gwangju 61469, Republic of Korea

**Keywords:** omega-3 fatty acid, heart arrest, cardiovascular mortality, meta-analysis

## Abstract

**Background/Objectives**: Sudden cardiac death (SCD) poses a significant burden on the modern-day public health system; however, while our understanding of the underlying pathophysiology is still evolving and may not be complete, many insights are known and applied every day. Targeted prevention methods are continually being developed and refined. We conducted a systemic review and meta-analysis to identify a blood nutritional biomarker that can predict and screen population groups at high risk for cardiovascular disease mortality (CVD mortality) or SCD. **Methods**: The literature search was conducted from November 2023 to 31 January 2024. Based on previous literature research, we studied the association between omega-3 fatty acids (n-3 FA; eicosapentaenoic acid [EPA], docosapentaenoic acid [DPA] and docosahexaenoic acid [DHA]) and SCD and/or CVD mortality individually and in combination. We evaluated and selected 10 prospective cohort studies out of 1789 related publications, with an average follow-up period of 8.7 years. A multivariate adjusted hazard ratio (HR) with 95% confidence interval (CI) was calculated and sub-analyzed to obtain a general trend of reduced risk of SCD in a high n-3 FA intake group from the general population. **Results**: Finally, we included 10 articles with a total sample size of 310,955 participants. We found an inverse association between circulating n-3 FA levels and SCD. The summary HR of SCD and CVD mortality for high versus low circulating n-3 FA levels (EPA + DHA + DPA) in serum plasma phospholipid was 0.55 (95% CI: 0.37–0.82) and that of EPA + DHA in RBC was 0.67 (95% CI: 0.45–0.99). Based on the sub-analysis, the HR of EPA (%) was 0.79 (95% CI: 0.60–0.82) and that of DHA (%) was 0.72 (95% CI: 0.60–0.87). **Conclusions**: Our results suggest a potential cardio-protective association between high EPA and DHA levels in blood and a reduced incidence of adverse cardiac events.

## 1. Introduction

Cardiovascular disease (CVD) accounts for 30% of global mortality and approximately 17 million deaths annually. Of all CVD-related deaths, approximately 40–50% are attributed to sudden cardiac deaths (SCDs), of which 80% is caused by ventricular tachyarrhythmia [1]. By definition, SCD refers to an unexpected natural death from a cardiovascular cause within a short time period, generally <1 h from symptom onset, in a person without any prior fatal condition [2]. Although the survival rate among patients with sudden cardiac arrest (SCA) is increasing owing to significant advances in cardiopulmonary resuscitation (CPR) and post-resuscitation care, it remains poor, at less than 1% worldwide and 5% in the United States [3]. A reported 40% of SCDs occur unwitnessed, most often at home [2], which further increases fatality. However, although >6 million SCDs occur annually [1], and the incidence continues to increase, an effective precursor to predict or prevent adverse cardiac events is lacking. Therefore, we conducted a meta-analysis to find a potential nutritional blood biomarker for SCA/SCD.

### Omega-3 Fatty Acids

The American Heart Association (AHA) first reviewed the potential benefits of omega-3 (n-3) polyunsaturated fatty acids (PUFAs) in 1996 [4], and their benefits in preventing ventricular arrhythmia and thus cardiovascular diseases (CVDs) have been reported by several consequent studies [5,6,7,8]. Omega-3 long-chain PUFAs (n-3 FA) are dietary fats incorporated in many parts of the body, including cell membranes, and they play a significant role in anti-inflammatory response and cell membrane viscosity [9]. The proposed mechanisms suggest that n-3 FA has modulating effects on arterial lipoprotein lipase levels that are linked to lipid deposition change in the arterial wall and local macrophage-mediated inflammatory process. An increasing number of studies report that n-3 FAs reduce the synthesis of pro-inflammatory cytokines and thus alleviate inflammations, which are considered the main cause of arteriosclerosis [10]. Considering this premise, several epidemiological studies and randomized controlled trials (RCTs) proved the effectiveness of n-3 FAs in preventing CVD [7,8,11]. In fact, the AHA recommends a daily consumption of 1 g of EPA + DHA for patients with documented coronary heart disease (CHD) and two fish per week for healthy individuals [12]. Plant-derived (a-linolenic acid; ALA, C18:3n-3) and marine-derived (eicosapentaenoic acid, C20:5n-3 [EPA] and docosahexaenoic acid, C22:6n-3 [DHA]) n-3 FAs differ; this paper will solely focus on the latter.

Despite the comprehensive research on the association between n-3 FA and CVD incidence, studies on the association between n-3 FA and the risks of SCD or CVD mortality are lacking. The first clinical evidence of the possible protective effect of n-3 FA on SCD was published in 1999 during the GISSI-Prevenzione trial [13]. However, most existing studies are RCTs that use n-3 FA as part of treatment after the initial cardiac event (i.e., acute myocardial infarction) [14] or measure dietary n-3 FA intake in the forms of supplement or oily fish [15,16]. We concluded that circulating n-3 FA levels can best represent cardiovascular health and predict the risks of adverse outcomes. Therefore, we decided to perform a comprehensive meta-analysis of a population cohort and observational studies that measured circulating blood n-3 FA levels to determine its possible association with SCD and/or CVD mortality risks. This study aimed to review previous studies on circulating n-3 FA levels in the general population and test its effectiveness in predicting SCD events.

## 2. Materials and Methods

The following meta-analysis was conducted in accordance with the Preferred Reporting Items for Systematic Reviews and Meta-Analyses (PRISMA) statement [17].

### 2.1. Data Source and Search

We conducted the literature search primarily on the PubMed and EMBASE databases. The search terms used were (“Omega-3 Fatty Acids”, “Omega-3”, “n-3 FA”, “Docosahexaenoic acid” OR “Eicosapentaenoic acid”) AND (“Cardiac arrest”, “Sudden Cardiac Death”, “Heart arrest”, Cardiovascular death”, “Cardiovascular mortality”, “Cardiac death” OR “Sudden death”). No restrictions on the publication year were imposed, but only studies published up to August 2018 were reviewed. We intentionally focused only on observational studies to reflect real-world settings and excluded randomized controlled trials (RCTs) from our analysis. This decision was made to assess the association between naturally occurring circulating omega-3 fatty acid levels and clinical outcomes such as SCD and CVD mortality, which cannot be fully captured in fixed-dose intervention trials.

### 2.2. Inclusion Criteria

The inclusion criteria were (1) a population-based cohort or observational study design; (2) study population including adults aged >18 years who did not undergo cardiac surgery or were pregnant; (3) the exposure of interest was circulating n-3 FA levels (EPA, DHA, DPA); (4) the end points or main outcomes were SCA, SCD or CVD mortality; (5) adjusted hazard ratios (HRs) or relative risk (RR) with corresponding 95% confidence intervals (CIs) were reported; and (6) the language of reporting was English. When more than one study studied the same population, the study that provided the most detailed data was selected.

### 2.3. Data Extraction

Two reviewers (JYK and SYK) independently screened all references and systematically excluded studies based on the title and abstract. Following this initial screening process, they conducted a full-text review of studies deemed eligible. Data were collected using standardized data extraction forms. Any discrepancies between the two reviewers were resolved through consensus meetings. The extracted information included the last name of the first author, year of publication, cohort name, country, mean or median age of participants at baseline, sample size, follow-up duration (years) and the adjusted hazard ratios (HRs) with corresponding 95% confidence intervals (CIs) for comparisons of the lowest versus highest circulating levels of EPA and DHA. The HRs adjusted for the maximum number of confounding variables were selected for this meta-analysis. The quality of the included studies was evaluated using the National Institute of Health (NIH) Quality Assessment Tool for Observational Cohort and Cross-Sectional Studies (https://www.nhlbi.nih.gov/health-topics/study-quality-assessment-tools, accessed on 13 December 2024).

### 2.4. Omega-3 Index

Conventionally, n-3 FA levels have been measured as plasma phospholipid levels and quantified as a percentage of total fatty acids [18]. In 2004, Harris and von Schacky first introduced the “omega-3 index” as a new biomarker and risk factor to indicate the content of EPA + DHA in red blood cell (RBC) membranes (as a percentage of total FA), as RBC membranes reflect cardiac membrane n-3 FA content [19]. Since then, most studies have almost exclusively used the omega-3 index. This meta-analysis thus contains two different measures of n-3 FA, both in RBC and plasma phospholipids. Yet, phospholipids remain as the biomarkers of longer term (4–8 weeks) circulating FA levels with similar response levels as in erythrocyte membranes [18]; thus, the differentiation would have negligible impact in understanding the general trend.

### 2.5. Statistical Analysis

We calculated the pooled estimate of adjusted hazard ratios (HRs) and their corresponding 95% confidence intervals (CIs) using a random-effects model, as this approach yielded more conservative results [20]. Heterogeneity among the included studies was assessed using the Cochran Q statistic, while the degree of variability was quantified using the *I*^2^ statistic. Heterogeneity was categorized as follows: *I*^2^ < 25% indicated low heterogeneity; 25% ≤ *I*^2^ ≤ 75% indicated moderate heterogeneity; and *I*^2^ > 75% indicated high heterogeneity [21]. To assess publication bias, we utilized funnel plots and the Egger regression model, applying a significance level of 10%, as previously recommended [22,23]. In cases where publication bias was detected, the Duval and Tweedie trim-and-fill method was applied as a sensitivity analysis to evaluate the impact of potentially missing studies on the overall pooled estimates. We used the DerSimonian–Laird (DL) estimator for the random-effects model in our meta-analysis to pool the hazard ratios (HRs) and corresponding 95% confidence intervals (CIs). The DL method was chosen due to its widespread use and simplicity in handling between-study variance.

We used Comprehensive Meta-Analysis (CMA) version 3 (Englewood, NJ, USA) to analyze the data, compute and plot the pooled HRs with 95% CIs for the lowest versus highest n-3 FA levels. Most HRs in the reviewed studies were matched based on age, sex and pre-existing medical conditions; yet, two were calculated based on the crude ratio.

## 3. Results

### 3.1. Literature Search

**Figure 1:**
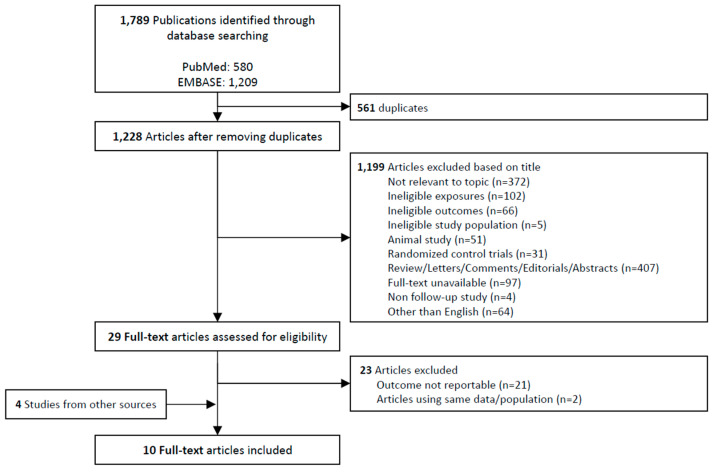
Flowchart of study selection (PRISMA)**.**

A total of 1789 publications were identified from the initial database search. After excluding 561 duplicates, the remaining 1199 articles were assessed based on the title and abstract. We performed full-text assessments of 29 articles for eligibility; 23 articles were excluded due to unreportable outcomes (*n* = 21, mostly the lack of endpoint data value and duplicative data/population [*n* = 2]), and 6 articles were included for meta-analysis [24,25,26,27,28,29]. However, because of the small sample size, additional literature search was conducted, and 4 other articles were selected for final inclusion [14,30,31,32], yielding 10 full-text articles. Figure 1 shows the flowchart of study selection.

### 3.2. Study Characteristics

Overall, the studies included a total of 15,783 participants with 2279 SCD and CVD mortality cases. Four studies measured EPA + DHA + DPA levels in plasma phospholipids [24,25,29,32], and three studies measured either the omega-3 index (EPA + DHA) or EPA + DHA content in the RBC membrane [14,27,28]. Given that a standard for measuring EPA or DHA levels does not exist, the analysis was conducted separately for each RBC membrane n-3 FA or with omega-3 index and plasma phospholipid for distinction. However, the general trends across the two sub-analyses remain consistent.

**Figure 2:**
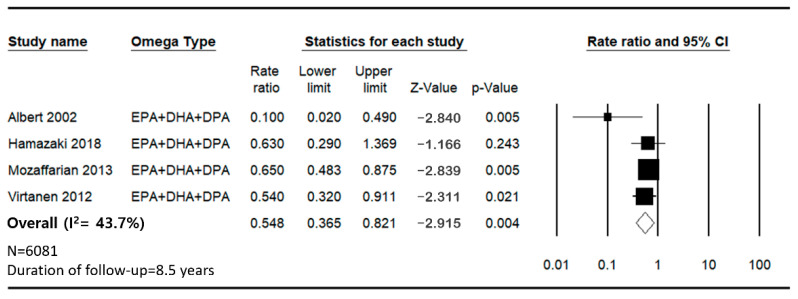
Forest plot for sudden cardiac death and cardiovascular disease mortality for the lowest versus highest categories of total n-3 fatty acids (EPA + DHA + DPA) in plasma phospholipid [24,25,29,32].

The pooled analysis from the meta-analysis indicates a significant inverse association between the intake of n-3 FAs (EPA + DHA + DPA) and the incidence of SCD, with a combined HR of 0.548 (Figure 2).

This indicates a 45.2% reduction in the risk of SCD among individuals with higher intake of these fatty acids. The heterogeneity among the studies was moderate, with an *I*^2^ value of 43.73%, which implies that although the overall effect was consistent, variations attributable to different study populations, dosages or methods were present.

### 3.3. Sub-Group Meta-Analysis

**Figure 3:**
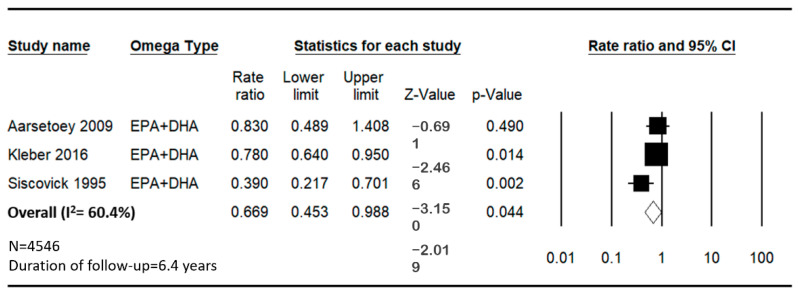
Forest plot for sudden cardiac death and cardiovascular disease mortality for the lowest versus highest categories of omega-3 index (EPA + DHA) in red blood cell membrane [14,27,28].

The pooled analysis from the meta-analysis also indicates a significant inverse association between the intake of EPA + DHA and the incidence of SCD, with a combined HR of 0.669. The heterogeneity of the studies was moderate, with an *I*^2^ value of 60.36% (Figure 3).

**Figure 4:**
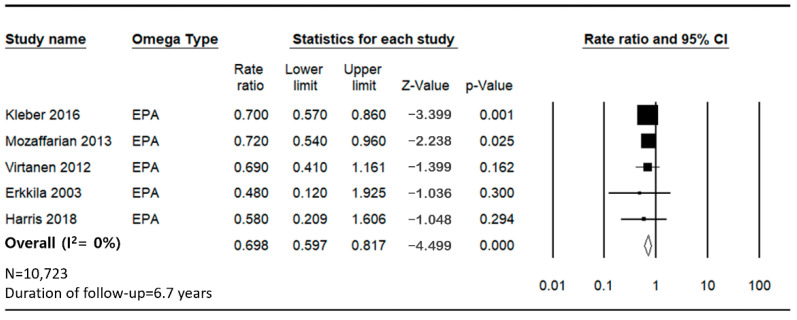
Forest plot for sudden cardiac death and cardiovascular disease mortality for the lowest versus highest categories of EPA [27,29,30,31,32].

The pooled analysis indicates a significant inverse association between the intake of EPA and the incidence of SCD, with a combined HR of 0.698. The heterogeneity among the studies was moderate, with an *I*^2^ value of 10.24% (Figure 4).

**Figure 5:**
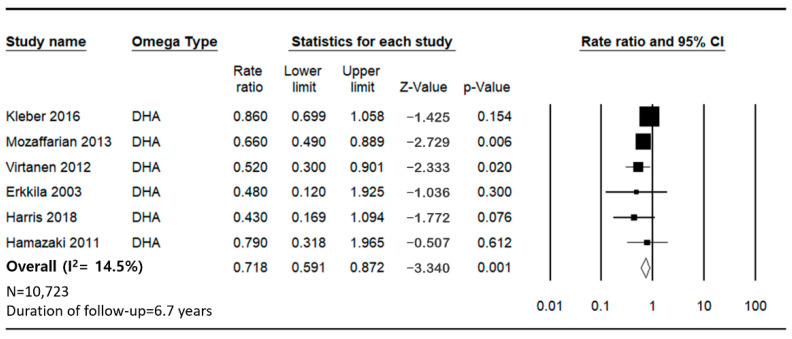
Forest plot for sudden cardiac death and cardiovascular disease mortality for the lowest versus highest categories of DHA [27,29,30,31,32].

The pooled analysis indicates a significant inverse association between the intake of DHA and the incidence of SCD, with a combined HR of 0.718. The heterogeneity of the studies was moderate, with an *I*^2^ value of 14.53% (Figure 5).

## 4. Discussion

The findings from this meta-analysis and systematic review provide evidence for a strong inverse association between n-3 FAs and SCD. The results are consistent with existing findings that n-3 FAs have cardio-protective effects in healthy and unhealthy adults. Furthermore, the cumulative HR indicates that n-3 FAs also reduce the risk of SCD in the normal population.

To our knowledge, this is the first study to investigate the association between circulating n-3 FA levels and the risk of SCD. Previous studies on this subject mostly assessed the benefits of the administration of a regular dose of dietary n-3 FA as part of clinical treatment, if not secondary prevention, in patients with pre-existing medical history in randomized controlled settings [33,34]. Therefore, the actual clinical application of those findings in the general patient population is limited. In contrast, given that the studies we included were observational, we could safely generalize that high EPA + DHA levels in blood may have a protective effect on SCD or other adverse cardiovascular events in a real-world setting. Therefore, our study is significant in providing evidence for the use of blood n-3 FA levels as nutritional biomarkers, if not a predictor, of SCA/SCD to screen high-risk populations for primary prevention.

Several previous systematic reviews and meta-analyses have evaluated the association between omega-3 fatty acid (FA) intake and cardiovascular disease (CVD) outcomes, particularly CVD mortality and sudden cardiac death (SCD). One study conducted a meta-analysis of 10 randomized controlled trials (RCTs) involving 77,917 individuals and found that omega-3 FA supplementation modestly reduced the risk of cardiovascular events [35]. Another study reported a significant reduction in major cardiovascular events with omega-3 FA intake, particularly EPA and DHA. However, some studies did not observe statistically significant benefits of omega-3 supplementation for reducing major cardiovascular events [36,37]. Other studies highlighted the role of omega-3 FAs in preventing SCD among coronary heart disease (CHD) patients [38,39], while another reported that omega-3 FA supplementation demonstrated effects comparable to statins in reducing all-cause and cardiovascular mortality [40]. These discrepancies among studies may be attributed to differences in study design, study populations and outcome measures.

Unlike these previous studies, our analysis specifically focused on prospective cohort studies evaluating circulating blood omega-3 FA levels and their association with SCD and CVD mortality. While earlier studies primarily assessed the effects of omega-3 supplementation, we aimed to examine naturally occurring circulating omega-3 FA levels to reflect real-world conditions more accurately. This approach offers a more generalized understanding of the predictive value of blood omega-3 FA levels, as opposed to fixed-dose interventions in controlled RCTs. Therefore, our study provides essential evidence for the utility of blood omega-3 FAs as potential biomarkers for SCD and CVD mortality prediction, contributing practical and broadly applicable insights for primary prevention strategies.

However, the relationship between n-3 FAs and fatal arrhythmias, which are major contributors to SCD, warrants further discussion. While n-3 FAs are known to stabilize cardiac cell membranes and reduce inflammation, their effects on suppressing ventricular tachy-arrhythmias remain complex and not fully understood [41]. Studies have suggested that n-3 FAs may influence ion channels, particularly sodium and calcium channels, thereby reducing the occurrence of arrhythmias [42,43]. However, the molecular mechanisms involved are intricate and may vary depending on individual genetic predispositions, comorbid conditions and circulating levels of n-3 FAs [44]. Future research should delve deeper into these pathways to clarify the mechanistic basis of the anti-arrhythmic effects of n-3 FAs. This understanding could aid in developing targeted nutritional or pharmacological interventions for SCD prevention.

These research findings further support the strong association between the intake of n-3 FAs, particularly EPA and DHA, and the reduction in SCD risk. These findings improve our understanding of the positive effect these specific types of n-3 FAs have on cardiovascular health, offering important information for devising nutritional strategies to improve heart health. The heterogeneity among studies indicates variations attributable to differences in study populations, dosages or methods, highlighting the need for more precise guidelines on the optimal intake of n-3 FAs for specific populations or the method of intake.

We did not conduct a cumulative sub-analysis based on study population, as only one or two studies included participants with CVD or CKD, respectively. Two studies targeted a population with CVD, specifically with acute coronary syndrome [14] and coronary artery disease [30]. However, the statistical data from individual studies show that among participants with CKD undergoing hemodialysis, a high n-3 FA intake did not yield a reduced risk of SCD [26]. However, among participants with CVD, similar trends of decreasing HRs as in the general population were observed. The HRs differed slightly across different sub-groups of the population with CVD. From studies that included both CHD and SCD as CVD mortality, the decrease in HR with high n-3 FA intake was greater for SCD than for CHD [25].

This study emphasizes the importance of a nutritional approach in the prevention and management of heart disease, with the potential to influence public health policies and clinical guidelines. Recommending an increase in the intake of n-3 FAs could be an effective approach to reducing the risk of CVDs, including SCD, serving as a crucial strategy for improving population health.

### Limitations

This study has a few limitations owing mainly to the low incidence of SCD. Given the small sample size, the criteria we established further decreased the number of eligible studies. In fact, several studies were published in Europe, particularly in regions around the Mediterranean Sea, and Japan that reported high fish consumption among participants; however, most of these studies were in local languages and thus had to be excluded. In addition, although the study aimed to understand the general trend and association, we could not definitively determine the association between RBC and plasma PL levels of EPA and DHA and CVD risk. A study reported up to a 3.5-times difference in erythrocyte EPA + DHA level measurements across laboratories and research institutions owing to differences in their analysis methods [45], which raises some concern. Thus, further research is needed to definitely prove the protective effect of n-3 FAs and their use as accurate and reliable nutritional biomarkers of SCA or SCD. Finally, we acknowledge that a formal risk of bias assessment was not conducted for the included observational studies. This is a limitation of our study. In future analyses, we will utilize appropriate tools, such as the Risk of Bias in Non-Randomized Studies of Exposures (ROBINS-E) tool, to ensure a comprehensive assessment of bias in observational studies.
